# Development and validation of an interpretable ultrasound radiomics model for benign and malignant classification of breast lesions: a multicenter large-sample study

**DOI:** 10.1186/s13244-026-02344-y

**Published:** 2026-06-25

**Authors:** Di Zhang, Wen-Wu Lu, Xia-Chuan Qin, Wang Zhou, Xian-Ya Zhang, Yan-Hong Luo, Lin-Song Wu, Jun Wu, Jun-Li Wang, Jun-Jie Zhao, Lei Zhang, Chao-Xue Zhang

**Affiliations:** 1 Department of Ultrasound, The First Affiliated Hospital of Anhui Medical University, 230022 Hefei, Anhui China; 2 Department of Medical Ultrasound, Chengdu Second People’s Hospital, 610000 Chengdu, Sichuan China; 3 Department of Medical Ultrasound, Tongji Hospital, Tongji Medical College, Huazhong University of Science and Technology, 430030 Wuhan, Hubei China; 4 Department of Medical Ultrasound, The Third Affiliated Hospital of Anhui Medical University, Hefei First People’s Hospital, 230061 Hefei, Anhui China; 5 Department of Ultrasound, Fu Yang People’s Hospital, 236000 Fuyang, Anhui China; 6 Department of Ultrasound, The Second Affiliated Hospital of Anhui Medical University, 230601 Hefei, Anhui China; 7 Department of Ultrasound, WuHu Hospital, East China Normal University (The Second People’s Hospital, WuHu), 241001 Wuhu, Anhui China; 8 Department of Medical Ultrasound, Fuyang Cancer Hospital, 236000 Fuyang, Anhui China; 9 Department of Medical Ultrasound, Huainan Oriental Hospital Group General Hospital, 232000 Huainan, Anhui China

**Keywords:** Ultrasound, Breast cancer, Machine learning, Radiomics, BI-RADS

## Abstract

****Objectives**:**

To develop and validate a combined ultrasound-based radiomics-clinical model for differentiating benign and malignant breast lesions.

****Materials and methods**:**

A total of 3142 patients from eight hospitals between February 2012 and September 2024 were included in this multicenter retrospective development and validation study, with an additional single-center prospective test cohort. Lesions were manually segmented, and radiomics features were automatically extracted to construct five machine learning models. The best-performing radiomics model was combined with clinical features to build a combined model. Model performance and its impact on Breast Imaging Reporting and Data System (BI-RADS)-based biopsy decisions were evaluated.

****Results**:**

Logistic regression (LR) showed the best radiomics performance, with area under the curves (AUCs) of 0.83, 0.82, 0.81, and 0.82 across the training, internal test, external test, and prospective test sets. The clinical model achieved AUCs of 0.87, 0.85, 0.87, and 0.86, whereas the combined model achieved AUCs of 0.92, 0.90, 0.92, and 0.93, significantly outperforming both single-modality models (all *p* < 0.01). Decision curve analysis (DCA) showed that the combined model had a higher net benefit than the other models across a broad range of threshold probabilities (0.05–0.95) in this study. Performance remained stable across lesion size and age subgroups. In the reclassification analysis, the model suggested the potential to influence biopsy recommendations without a significant reduction in sensitivity and to increase the malignancy yield in BI-RADS 4a. Shapley additive explanations (SHAP) analysis provided clinically interpretable feature contributions.

****Conclusion**:**

The interpretable ultrasound-based radiomics model enables reliable, noninvasive breast lesion diagnosis and may reduce unnecessary biopsies.

**Critical relevance statement:**

This work developed an interpretable radiomics-clinical combined model in a multicenter retrospective development and validation study, with additional testing in a single-center prospective cohort, and may support breast lesion risk stratification and biopsy decision-making after further prospective clinical utility evaluation.

****Key Points**:**

- Conventional ultrasound diagnosis of breast cancer shows limited specificity.
- A multicenter radiomics-clinical combined model showed improved diagnostic performance, with additional validation in a prospective test cohort.

**Graphical Abstract:**

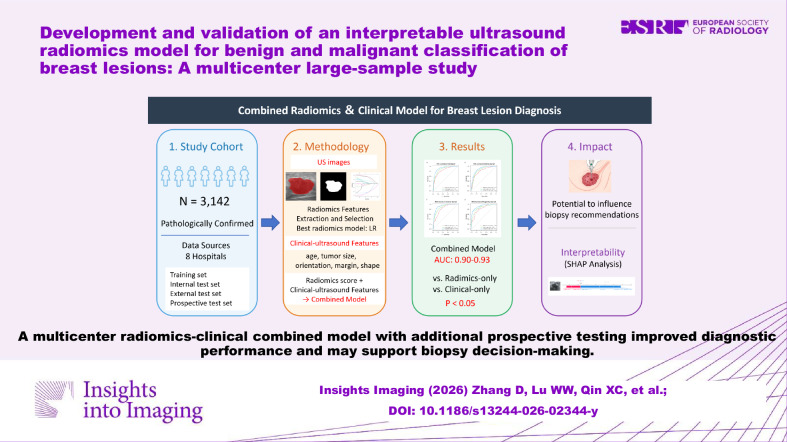

## Introduction

Breast cancer has become the most common malignancy and the leading cause of cancer-related mortality in women globally [1]. Early detection and accurate diagnosis are crucial for guiding treatment and improving outcomes [2, 3]. Common imaging modalities for breast lesion evaluation include mammography, ultrasound, and magnetic resonance imaging (MRI) [4]. Mammography is effective for early detection but has limited sensitivity in women with dense breasts [5]. MRI offers high resolution but is restricted by cost and accessibility [6].

Ultrasound, as a real-time and radiation-free imaging modality, is widely used for the initial screening and diagnosis of breast lesions [7, 8]. However, the interpretation of ultrasound images is highly dependent on the experience of radiologists, introducing a degree of subjectivity that may lead to inconsistencies in diagnosis. To address this issue, the American College of Radiology introduced the fifth edition of the Breast Imaging Reporting and Data System (BI-RADS) in 2013 [9]. Although BI-RADS has improved objectivity, distinguishing atypical benign findings from malignant lesions remains difficult due to overlapping imaging features. In particular, when breast lesions are evaluated using B-mode ultrasound (BMUS) alone, category 4 lesions show high false-positive rates and lack detailed sub-classification criteria [10–13], leading to frequent reliance on biopsy. Although incorporating mammography, color Doppler, and relevant clinical information may further improve lesion assessment, biopsy remains common and increases patient burden, highlighting the need to improve diagnostic accuracy while reducing unnecessary interventions.

Radiomics is a new analytical method that allows for the extraction of numerous quantitative features from medical images, helping to reveal subtle pathological information hidden within the imaging data [14, 15]. Radiomics has been widely applied in breast disease, especially when combined with ultrasound imaging [16–18]. Previous studies have leveraged machine learning algorithms to develop models for accurate classification of breast lesions [19–22]. However, most existing studies only focused on single machine learning methods and lacked prospective, multicenter, large-scale validation, limiting the generalizability and clinical applicability. Moreover, few studies have specifically focused on reducing unnecessary biopsies in clinical practice, and comprehensive evaluations of the clinical utility of these models remain limited.

Therefore, this study aims to develop and validate an integrated radiomics model combining ultrasound-based features with clinical-ultrasound characteristics. The model’s performance will be tested across multicenter and prospective datasets, with particular emphasis on its impact on BI-RADS classification and biopsy recommendations. To improve interpretability, Shapley additive explanations (SHAP) will be used to clarify the model’s decision-making process.

## Materials and methods

This study received approval from the Institutional Ethics Committee, with separate approval numbers for the retrospective (No. PJ2023-07-11) and prospective (No. PJ2024-02-12) components. The informed consent was waived for the retrospective analysis due to its design, whereas all participants enrolled in the prospective phase provided written informed consent.

### Research subjects

Patients with breast lesions from eight tertiary hospitals were enrolled in this study, including a retrospective cohort between February 2012 and March 9, 2024, and a prospective cohort at Hospital 1 between March 10 and September 2024. The inclusion criteria were as follows: (1) age ≥ 18 years; (2) breast lesions identified by ultrasound; (3) breast ultrasound performed within 2 weeks before biopsy or surgery. For the retrospective cohort, pathological confirmation of the lesion was required, whereas for the prospective cohort, patients were enrolled when biopsy or surgical treatment was planned and were included in the final analysis only if pathology was obtained. The exclusion criteria were: (1) prior biopsy of the target lesion before ultrasound examination, or a history of chemotherapy, radiotherapy, or other malignancies; (2) incomplete clinical or pathological data; (3) incomplete or poor-quality ultrasound images. In the prospective cohort, patients who did not ultimately undergo biopsy or surgery were also excluded. The patient enrollment process is illustrated in Supplementary Fig. S1.

In total, this study included data from 3142 eligible patients with breast lesions from eight hospitals for model training and testing. Among them, 2412 patients from three hospitals in the retrospective cohort were randomly divided into a training set (1688 patients) and an internal testing set (724 patients) in a 7:3 ratio. Additionally, 463 patients from the remaining five hospitals were included in a combined external testing set. Furthermore, 267 patients from Hospital 1 in the prospective cohort were included as the prospective testing set.

### Clinic-pathologic data and ultrasound image collection

Ultrasound images were acquired using high-frequency linear-array transducers on the following systems: Resona 5S/6S/7S/7T/8, DC-8, and Nuewa R9 (Mindray), VOLUSON E8 and LOGIQ E9 (GE), iU22 and EPIQ 5/7/7 C (Philips), WS80A and RS80A (Samsung), ACUSON S2000 and ACUSON Sequoia (Siemens), AixPlorer (SuperSonic Imaging), MyLab 9 (Esaote), and ARIETTA 70 (Hitachi). During image acquisition, depth, gain, and focal zone were optimized to ensure adequate image quality, and the maximum lesion diameter was recorded. Representative images capturing key features of each breast mass were stored in the picture archiving and communication system (PACS) for subsequent analysis and validation.

Two senior radiologists with over 15 years of experience in breast ultrasound independently reviewed all ultrasound images without knowledge of pathology, assessing lesion features according to the fifth edition of BI-RADS. In the external and prospective test sets, they further classified lesions into BI-RADS categories 2, 3, 4a, 4b, 4c, and 5. Discrepancies were resolved by consensus. For patients with multiple ultrasound examinations, only the most recent scan within 2 weeks before biopsy/surgery was analyzed. The mean interval between the ultrasound examination and biopsy/surgery was 4.1 ± 2.9 days (range 0–13 days). The analysis was performed per patient, with one lesion included per patient. For multifocal lesions, only the largest lesion and its corresponding pathology result were included in the analysis.

### Image segmentation and feature extraction

The lesion segmentation process was initially performed by a radiologist A ( > 7 years of experience) using ITK-SNAP 3.8.0 software (http://www.itksnap.org). The region of interest (ROI) was manually delineated along the lesion boundary on the stored static ultrasound image showing the largest cross-section of each lesion. Radiomics features were then extracted automatically in Python 3.10.6 using the PyRadiomics package (version 3.1.0) [23]. Before feature extraction, image normalization (normalizeScale = 25) and resampling (ResampledPixelSpacing = [1, 1, 1]) were performed [24].

To evaluate reliability, 50 patients were randomly selected from the training set for re-segmentation by radiologist A and another radiologist B. Intraclass correlation coefficients (ICCs) were calculated, and features with ICC ≥ 0.80 were retained.

### Feature selection and machine learning classifier selection

Feature selection aimed to retain representative, stable, and clinically relevant features, as detailed in Supplementary Material S1. Based on the selected radiomics features, five commonly used machine learning classifiers were evaluated to identify the optimal predictive model. These classifiers included logistic regression (LR), Random Forest (RF), support vector machine (SVM), multi-layer perceptron (MLP), and K-nearest neighbor (KNN). Model performance was assessed by the area under the receiver operating characteristic (ROC) curve (AUC), accuracy, sensitivity, and specificity. The classifier with the best overall performance was chosen as the final radiomics model, and a radiomics score was calculated for each patient.

### Model development and performance evaluation

In the training set, univariate LR was used to identify clinical ultrasound risk factors for lesion malignancy. Variables with *p* < 0.1 were entered into multivariate LR. A stepwise backward selection approach was applied, and variables with *p* < 0.05 were selected to construct the clinical model. Additionally, a combined model was developed by integrating the radiomics score with clinical risk factors.

Decision curve analysis (DCA) was conducted to visualize the net clinical benefit of different models in guiding clinical decision-making. Net reclassification improvement (NRI) and integrated discrimination improvement (IDI) were calculated to quantify the incremental value of the combined model. Model calibration was evaluated using calibration curves and the Hosmer-Lemeshow test. Furthermore, subgroup analyses were performed according to lesion size and patient age.

### Clinical utility of the combined model in BI-RADS reclassification

Lesions in the external and prospective test sets were categorized by senior radiologists following BI-RADS recommendations: categories 2–3 as non-biopsy and categories 4–5 as biopsy. The combined model was applied to reclassify equivocal lesions: BI-RADS 4a lesions were downgraded to 3 if predicted low risk, and BI-RADS 3 lesions were upgraded to 4a if predicted high risk. Potential changes in biopsy recommendations after model-assisted BI-RADS reclassification and the malignancy proportion of BI-RADS 4a lesions were analyzed.

### Model interpretation and visualization

The SHAP method was incorporated into this study to enhance the interpretability of both the radiomics model and the combined model. Rooted in game theory, SHAP quantifies the contribution of each feature to the final prediction, providing a deeper understanding of the model’s decision-making process. Specifically, SHAP not only highlights the impact of individual features on different samples but also reveals interactions between features. By employing SHAP, physicians can better review and validate the model’s predictions, further ensuring its reliability and fairness in practical applications.

### Statistical analysis

Statistical analyses were performed using R software (version 4.2.2), MedCalc software (version 20.100), and SPSS software (version 24.0). The AUC values were compared using the DeLong test. Radiologists’ BI-RADS assessments were analyzed under three thresholds: (A) 4a, (B) 4b, and (C) 4c as cutoffs for malignancy. The McNemar test was used to compare the accuracy, sensitivity, and specificity between the combined model and radiologist assessments. A two-sided *p*-value < 0.05 was considered statistically significant.

## Results

### Study population and baseline characteristics

A total of 3142 patients with breast lesions were included, with a mean age of 49.1 ± 13.0 years (range, 18–93 years), comprising 1121 (35.6%) benign and 2021 (64.3%) malignant lesions. The mean ages of patients in the training, internal test, external test, and prospective test sets were 48.9 ± 13.0 years (range, 18–92 years), 48.9 ± 12.8 years (range, 18–85 years), 48.9 ± 13.4 (range, 18–83 years), and 51.0 ± 12.1 years (range, 23–93 years), respectively. Baseline clinical-ultrasound characteristics are summarized in Table 1.

**Table 1:** Clinical pathological characteristics of patients in the training set, internal test set, external test set, and prospective test set

Characteristic	Training set (*n* = 1688)	Internal test set (*n* = 724)	External test set (*n* = 463)	Prospective test set (*n* = 267)
Age, mean ± SD, years	48.9 ± 13.0	48.9 ± 12.8	48.9 ± 13.4	51.0 ± 12.1
Age				
< 40 years	396 (23.5)	162 (22.4)	114 (24.6)	42 (15.7)
≥ 40 years	1292 (76.5)	562 (77.6)	349 (75.4)	225 (84.3)
Tumor size, mean ± SD, cm	2.5 ± 1.2	2.6 ± 1.3	2.5 ± 1.4	2.6 ± 1.2
Tumor size				
≤ 2 cm	756 (44.8)	283 (39.1)	213 (46.0)	112 (41.9)
> 2 cm	932 (55.2)	441 (60.9)	250 (54.0)	155 (58.1)
Tumor location				
Left	917 (54.3)	364 (50.3)	228 (49.2)	138 (51.7)
Right	771 (45.7)	360 (49.7)	235 (50.8)	129 (48.3)
Orientation				
Parallel	1463 (86.7)	636 (87.8)	377 (81.4)	238 (89.1)
Nonparallel	225 (13.3)	88 (12.2)	86 (18.6)	29 (10.9)
Margin				
Well-circumscribed	704 (41.7)	292 (40.3)	153 (33.0)	67 (25.1)
Non-circumscribed	984 (58.3)	432 (59.7)	310 (67.0)	200 (74.9)
Echotexture				
Hypoecho	1487 (88.1)	635 (87.7)	402 (86.8)	211 (79.0)
Isoechoic	11 (0.7)	15 (2.1)	5 (1.1)	3 (1.1)
Hyperechoic	7 (0.4)	6 (0.8)	3 (0.6)	1 (0.4)
Complex cystic and solid	71 (4.2)	23 (3.2)	17 (3.7)	20 (7.5)
Heterogeneous	112 (6.6)	45 (5.2)	36 (7.8)	32 (12.0)
Shape				
Oval or round	350 (20.7)	148 (20.4)	111 (24.0)	50 (18.7)
Irregular	1338 (79.3)	576 (79.6)	352 (76.0)	217 (81.3)
Posterior features				
None	856 (50.7)	329 (45.4)	254 (54.9)	80 (30.0)
Shadowing	408 (24.2)	209 (28.9)	132 (28.5)	94 (35.2)
Enhancement	294 (17.4)	137 (18.9)	54 (11.7)	68 (25.5)
Combined pattern	130 (7.7)	49 (6.8)	23 (4.9)	25 (9.3)
Histologic type				
Benign	615 (36.4)	252 (34.8)	180 (38.9)	74 (27.7)
Fibroadenoma	350 (20.7)	148 (20.4)	114 (24.6)	31 (11.6)
Adenosis	159 (9.4)	64 (8.8)	33 (7.1)	22 (8.2)
Intraductal papilloma	19 (1.1)	11 (1.5)	6 (1.3)	4 (1.5)
Inflammatory lesion	41 (2.4)	17 (2.3)	12 (2.6)	13 (4.9)
Others	46 (2.8)	12 (1.8)	15 (3.3)	4 (1.5)
Malignant	1073 (63.6)	472 (65.2)	283 (61.1)	193 (72.3)
Invasive breast cancer	993 (58.8)	424 (58.6)	230 (49.7)	182 (68.2)
Non-invasive breast cancer	68 (4.0)	40 (5.5)	40 (8.6)	10 (3.7)
Others	12 (0.8)	8 (1.1)	13 (2.8)	1 (0.4)

### Radiomics feature selection and development of the radiomics model

A total of 851 radiomics features were extracted from each ROI. After reproducibility screening, redundancy filtering, and least absolute shrinkage and selection operator (LASSO)-based selection, 12 representative features were retained to build the radiomics model. The complete list of selected features is provided in Supplementary Material S2. Five machine learning classifiers were then trained with these features.

Although the RF model achieved an AUC of 1.00 in the training set, its performance decreased substantially in the internal, external, and prospective test sets (AUC = 0.74, 0.71, and 0.70), suggesting overfitting and limited generalizability. In contrast, the LR model maintained stable performance with AUCs of 0.83, 0.82, 0.81, and 0.82 across the four datasets. Importantly, the LR model significantly outperformed the other classifiers in all three test sets (all *p* < 0.001). Based on its robustness and generalizability, LR was selected as the final radiomics model, and a radiomics score was generated for each patient. The detailed performance metrics of all classifiers are provided in Table 2. Supplementary Fig. S2 illustrates the discriminative performance of the LR model.

**Table 2:** Diagnostic performance evaluation of five machine learning models in each dataset

	AUC (95% CI)	ACC	SEN	SPE	PPV	NPV
Training set						
KNN	0.78 (0.76–0.80)	80.98%	87.88%	68.94%	83.16%	76.53%
LR	0.83 (0.81–0.85)	76.48%	81.27%	68.13%	81.65%	67.58%
MLP	0.75 (0.73–0.77)	77.84%	86.21%	63.25%	80.36%	72.44%
RF	**1.00 (1.00–1.00)**^a^	100%	100%	100%	100%	100%
SVM	0.72 (0.70–0.75)	76.30%	86.30%	58.86%	78.54%	71.12%
Internal test set						
KNN	0.71 (0.67–0.74)	74.03%	81.57%	59.92%	79.22%	63.45%
LR	**0.82 (0.79–0.85)**^a^	76.66%	83.05%	64.68%	81.50%	67.08%
MLP	0.72 (0.69–0.75)	75.83%	84.32%	59.92%	79.76%	67.11%
RF	0.74 (0.70–0.77)	77.76%	87.29%	59.92%	80.31%	71.56%
SVM	0.73 (0.69–0.76)	76.66%	86.02%	59.13%	79.76%	69.30%
External test set						
KNN	0.67 (0.60–0.69)	69.11%	85.87%	42.78%	70.23%	65.81%
LR	**0.81 (0.78–0.85)**^a^	77.54%	90.64%	52.22%	75.50%	83.93%
MLP	0.73 (0.69–0.77)	76.03%	85.87%	60.56%	77.40%	73.15%
RF	0.71 (0.67–0.75)	73.65%	83.04%	58.89%	76.05%	68.83%
SVM	0.74 (0.70–0.78)	76.03%	84.10%	63.33%	78.29%	71.70%
Prospective test set						
KNN	0.67 (0.61–0.73)	73.41%	81.35%	52.70%	81.77%	52.00%
LR	**0.82 (0.77–0.87)**^a^	80.15%	94.82%	41.89%	80.97%	75.61%
MLP	0.68 (0.62–0.73)	79.03%	93.26%	41.89%	80.72%	70.46%
RF	0.70 (0.64–0.76)	80.90%	94.30%	45.95%	81.98%	75.56%
SVM	0.68 (0.62–0.73)	78.28%	91.71%	43.24%	80.82%	66.67%

Bold values indicate the model that achieved a significantly higher AUC than the other models within the corresponding dataset

*Acc* accuracy, *AUC* area under the receiver operating characteristic curve, *CI* confidence interval, *NPV* negative predictive value, *PPV* positive predictive value, *SEN* sensitivity, *SPE* specificity

^a^ Indicates that the AUC of this model is significantly higher than those of the other models according to the DeLong test (*p* < 0.05)

**Fig. 1:**
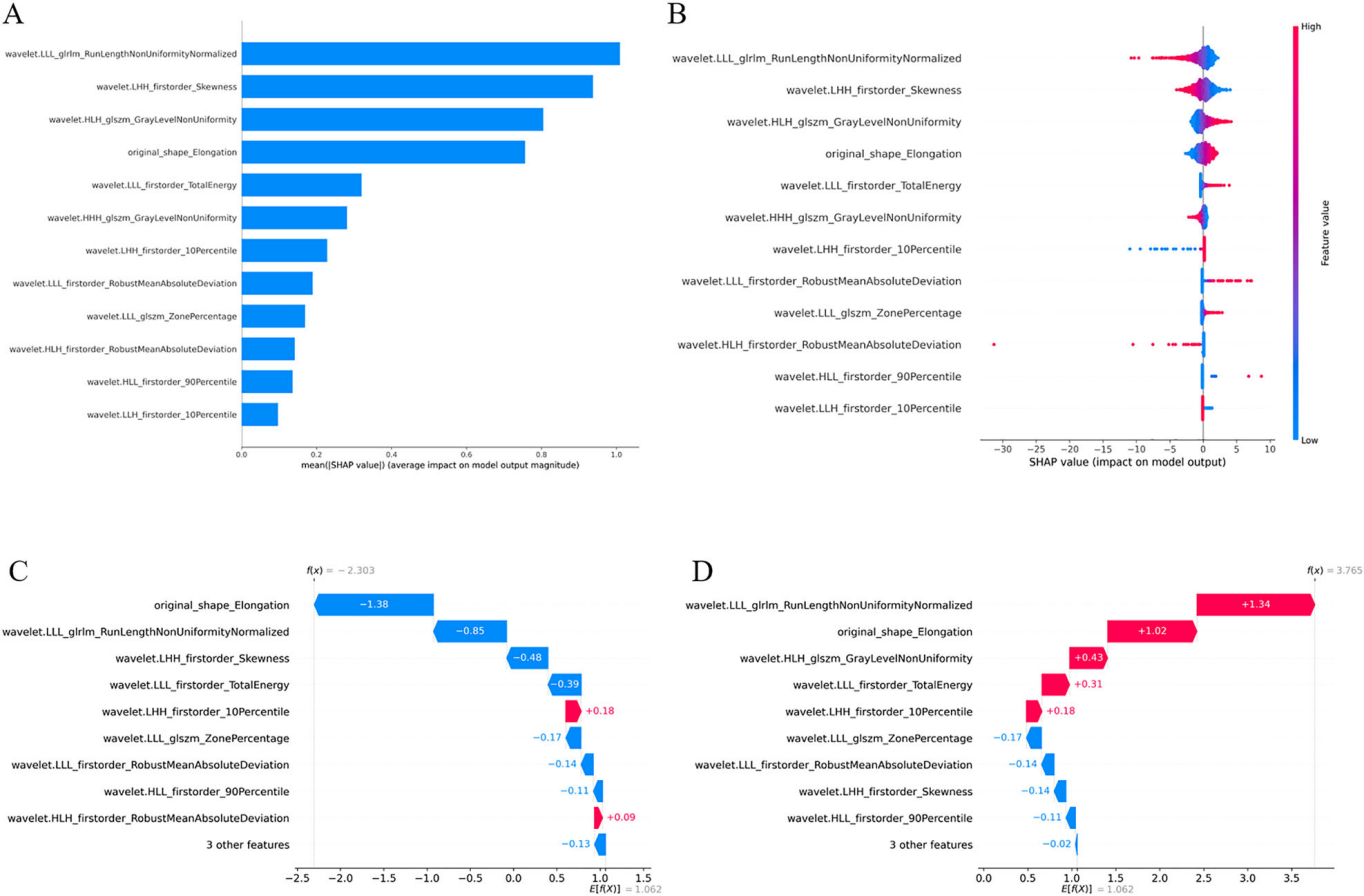
Feature importance and SHAP value visualization of the LR model. **A** Bar chart of average absolute SHAP values, ranking features by importance. **B** Beeswarm plot showing SHAP value distributions, with color indicating feature value (blue = low, red = high). **C**, **D** Waterfall plots for two patients illustrate feature contributions: red bars indicate positive, blue bars negative impacts. Patient **C** shows an overall negative prediction, while patient **D** shows an overall positive prediction

To enhance interpretability, SHAP analysis was applied to the LR model (Fig. 1), providing both global feature importance (bar and beeswarm plots) and case-level explanations (waterfall plots).

### Construction of the clinical model

Univariate LR of clinical-ultrasound features identified age, tumor size, orientation, margin, shape, echotexture, and posterior features as significant predictive factors for malignancy. Multivariate analysis further retained age, tumor size, orientation, margin, and shape as independent predictors. These five variables were used to construct the clinical model. The clinical model achieved an AUC of 0.87 in the training set, 0.85 in the internal test set, 0.87 in the external test set, and 0.86 in the prospective test set. Detailed regression results are shown in Supplementary Table S2.

### Construction and performance evaluation of the combined model

**Fig. 2:**
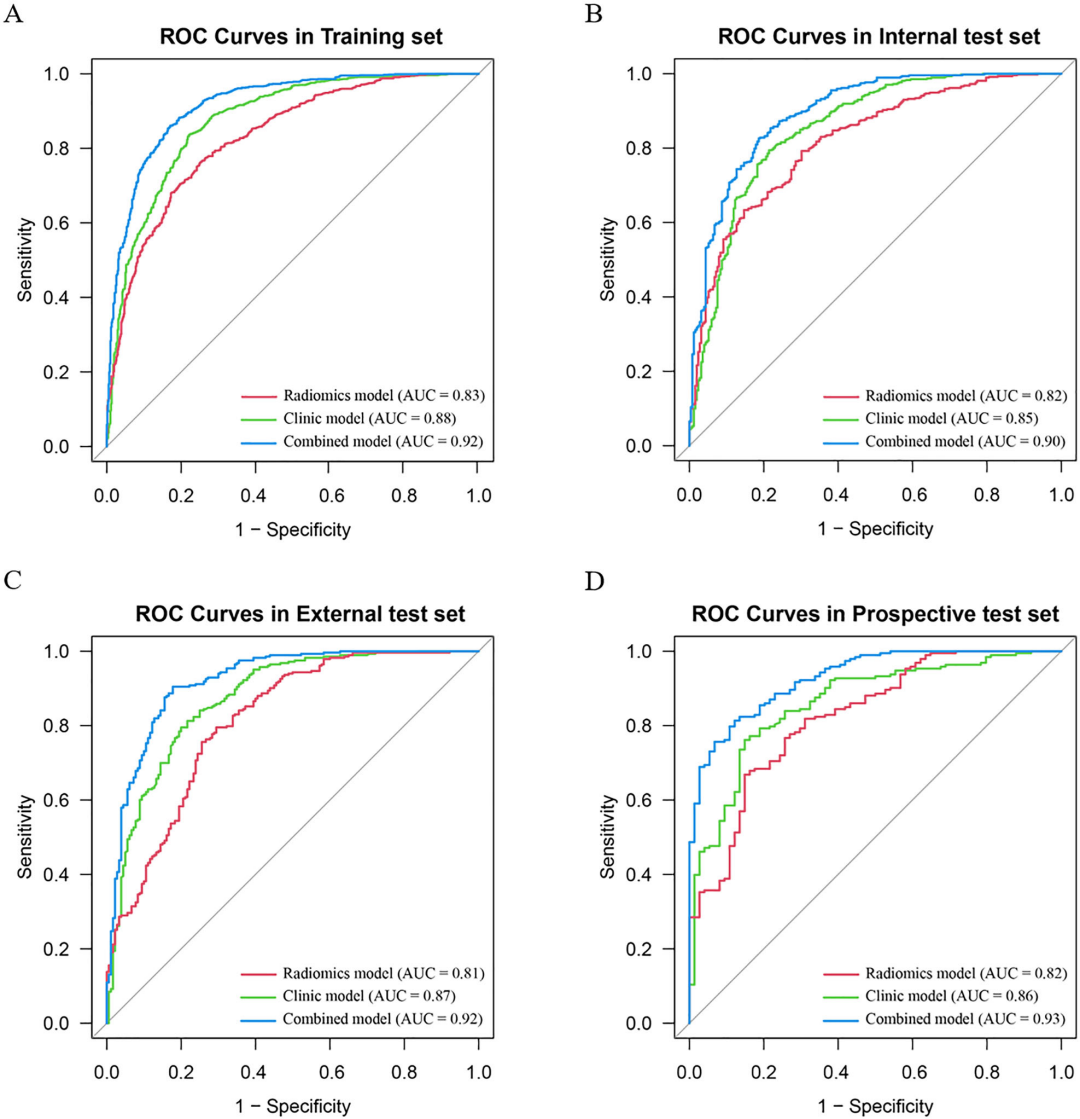
ROC curves of the radiomics model, clinical model, and combined model in different datasets. **A** training set; **B** internal test set; **C** external test set; **D** prospective test set

**Fig. 3:**
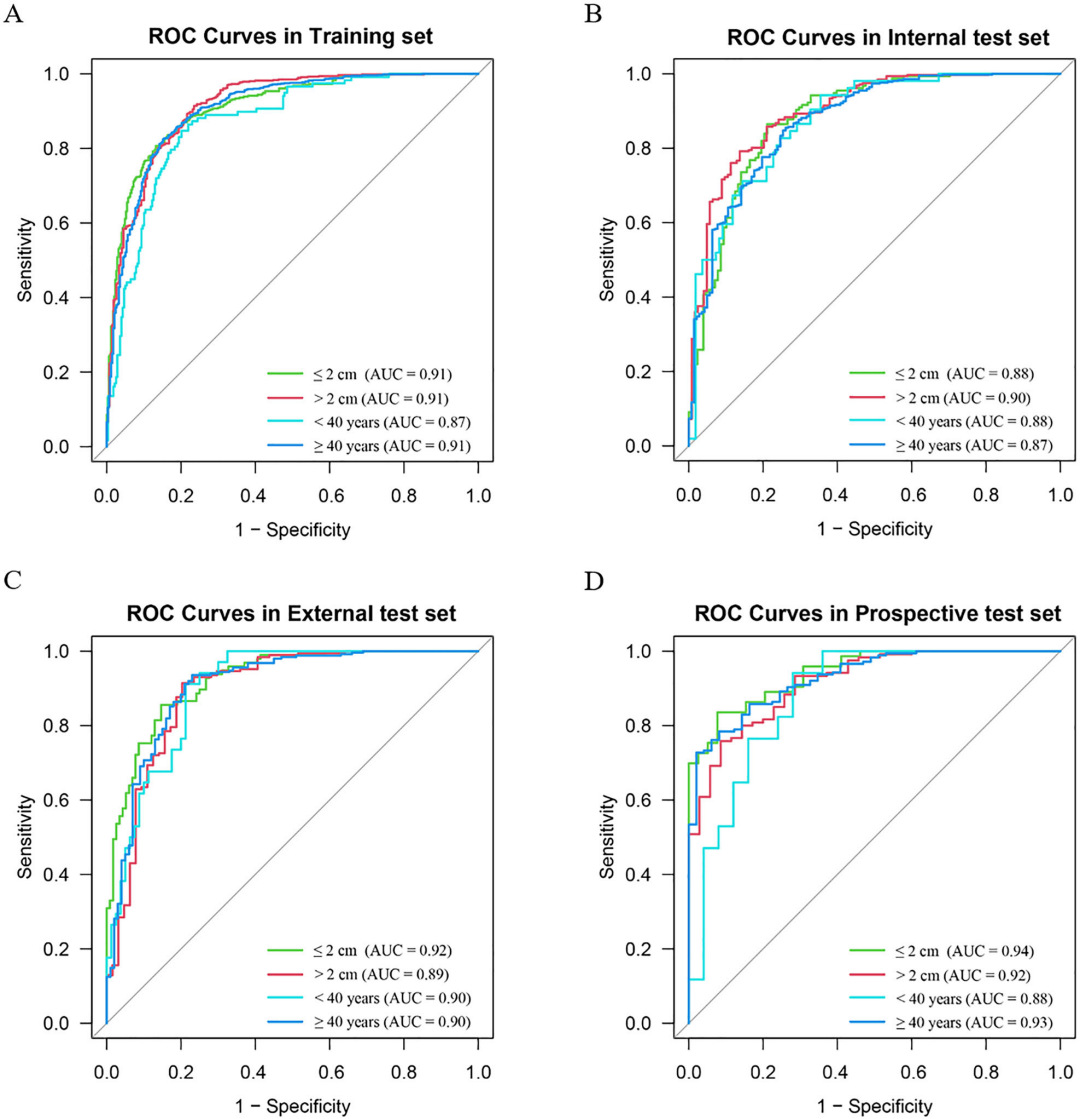
ROC curves of the combined model in different patient subgroups. **A** training set; **B** internal test set; **C** external test set; **D** prospective test set

A combined model was constructed by integrating the radiomics score with the five independent clinical-ultrasound predictors (age, tumor size, orientation, margin, and shape). In multivariate analysis, all six variables remained significant predictors of malignancy. The combined model achieved AUCs of 0.92, 0.90, 0.92, and 0.93 in the training, internal, external, and prospective test sets, respectively. Across all datasets, the combined model significantly outperformed both the radiomics-only and clinical-only models (all *p* < 0.05, DeLong test). The ROC curves comparing model performance are shown in Fig. 2, with detailed metrics summarized in Supplementary Table S3. The combined model demonstrated robust and balanced diagnostic performance across key patient subgroups, including patients with smaller lesions and younger age, achieving consistently high AUCs ranging from 0.87 to 0.94, along with balanced sensitivity and specificity across subgroups (Supplementary Table S4 and Fig. 3). This early presentation highlights the model’s reliability across clinically relevant subgroups.

**Fig. 4:**
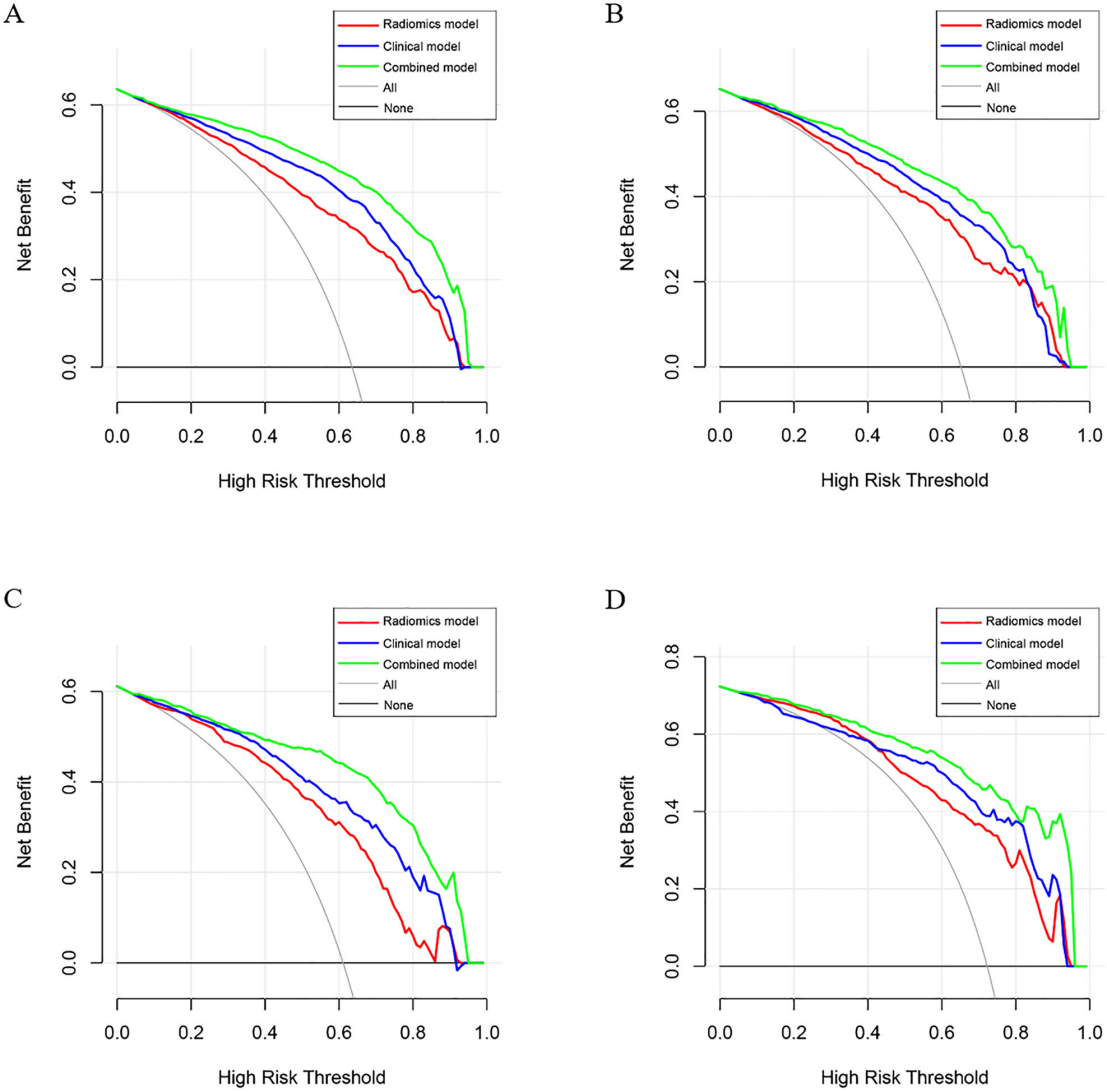
DCA curves of the radiomics model, clinical model, and combined model in different datasets. **A** training set; **B** internal test set; **C** external test set; **D** prospective test set

**Fig. 5:**
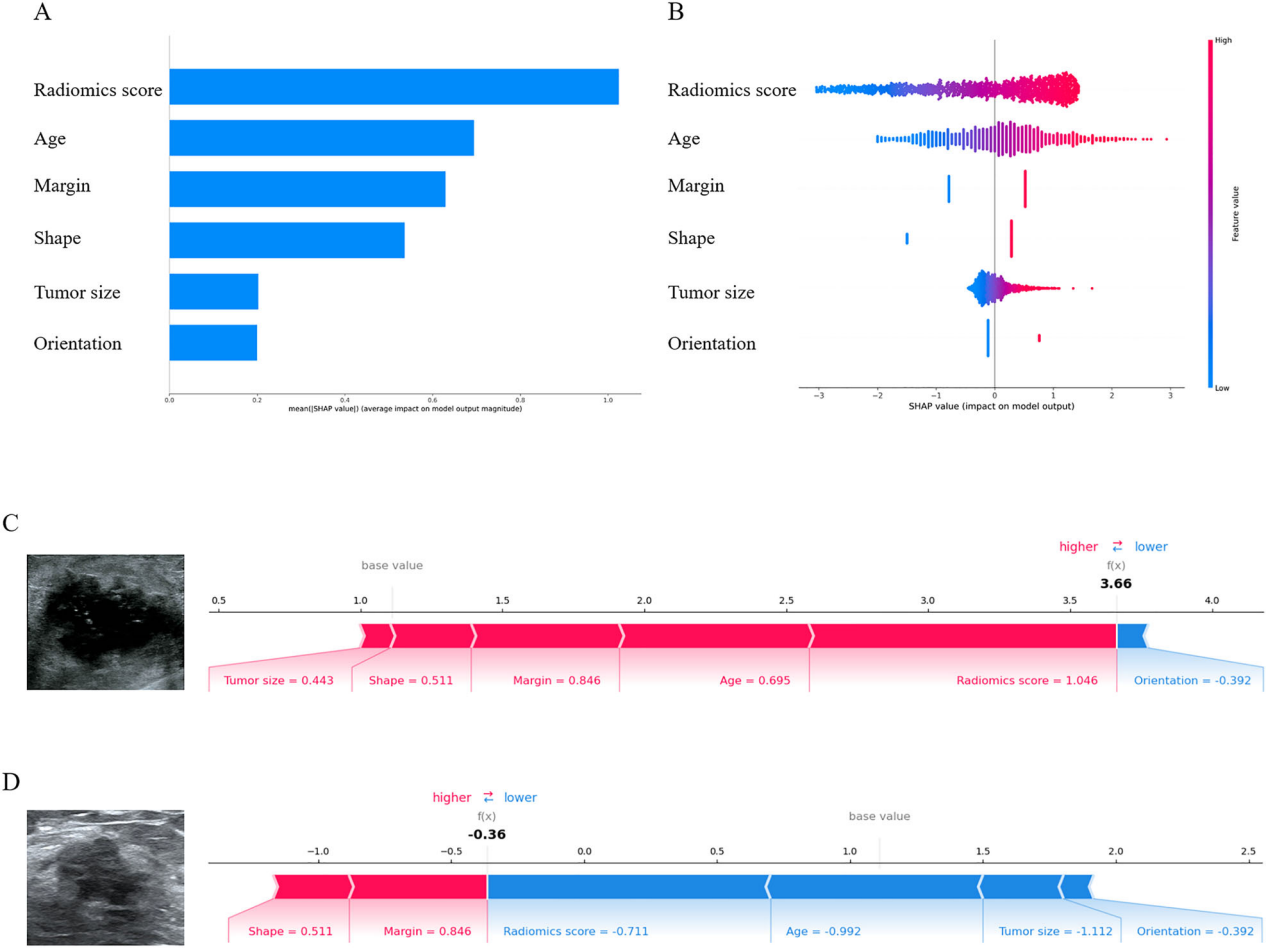
Feature importance and SHAP visualization of the combined model. **A** Bar chart ranking features by mean absolute SHAP values. **B** Swarm plot showing SHAP value distributions. **C**, **D** Case-specific waterfall plots with corresponding ultrasound images illustrate feature contributions. For patient **C** (*f*(*x*) = 3.66, invasive cancer), radiomics score, margin, shape, and tumor size contribute positively, while orientation is negative. For patient **D** (*f*(*x*) = −0.36, adenosis), margin and shape increase prediction, whereas age, radiomics score, tumor size, and orientation decrease it

The DCA showed that the combined model provided a higher net clinical benefit than either the radiomics or clinical model alone across a wide range of threshold probabilities (0.05–0.95) in all datasets (Fig. 4). Furthermore, both NRI and IDI analyses confirmed that incorporating the radiomics score significantly improved the discriminative ability of the clinical model (all *p* < 0.05; Supplementary Table S5). In addition, analysis of predicted risk probabilities demonstrated a clear distinction between benign and malignant breast lesions in all datasets, with malignant lesions showing markedly higher predicted probabilities (Supplementary Fig. S3). Model calibration was satisfactory, as demonstrated by calibration curves and Hosmer–Lemeshow tests across all datasets (Supplementary Fig. S4). SHAP visualizations provided global and individual-level interpretability by ranking feature importance and illustrating case-specific predictions (Fig. 5).

### Model performance compared with radiologists

In both the external and prospective test sets, we compared the diagnostic performance of the combined model with BI-RADS assessments made by experienced radiologists using different malignancy cutoff thresholds. When BI-RADS 4a was used as the cutoff, radiologist assessment achieved very high sensitivity but extremely low specificity. In contrast, the combined model significantly improved overall accuracy and specificity in both datasets, although its sensitivity was lower than that of BI-RADS 4. Using BI-RADS 4b as the cutoff, the combined model showed comparable overall accuracy to radiologist assessment, with significantly higher specificity but lower sensitivity. When BI-RADS 4c was applied, the overall diagnostic performance of the combined model and radiologist assessment was largely comparable, with no consistent significant differences in accuracy across datasets (Supplementary Table S6).

### Clinical utility of the combined model in BI-RADS reclassification

Using BI-RADS-based recommendations made by experienced radiologists, unnecessary biopsy rates were 29.67% (119/401) in the external test set and 24.21% (61/252) in the prospective test set. When biopsy recommendations were generated solely according to the combined model, the unnecessary biopsy rates were 10.22% (28/274) in the external test set and 10.47% (20/191) in the prospective test set, which were significantly lower than those based on BI-RADS recommendations (both *p* < 0.001).

Furthermore, the combined model was used to adjust BI-RADS 3 and 4a categories. After model-based reclassification, unnecessary biopsy rates decreased from 29.67% to 18.84% in the external test set (absolute reduction, 10.83%) and from 24.21% to 14.41% in the prospective test set (absolute reduction, 9.80%), without a significant reduction in diagnostic sensitivity. Notably, the malignancy rate among BI-RADS 4a lesions increased markedly after reclassification, from 9.23% to 44.44% in the external test set and from 16.67% to 50.00% in the prospective test set (both *p* < 0.05). Detailed results are summarized in Table 3.

**Table 3:** Performance of the combined model and BI-RADS classification in recommending biopsy for breast lesions

	Number of patients	Number of recommended biopsies	Benign lesions	Unnecessary biopsy rate	Malignancy rate of biopsy^a^
External test set					
Combined model	463	274	28	10.22%	-
BI-RADS classification	463	401	119	29.67%	9.23%
After BI-RADS classification adjustment	463	345	65	18.84%	44.44%
* P*^*1*^	-	-	-	< 0.001^b^	-
* P*^*2*^	-	-	-	< 0.001^b^	0.02^b^
Prospective test set					
Combined model	267	191	20	10.47%	-
BI-RADS classification	267	252	61	24.21%	16.67%
After BI-RADS classification adjustment	267	222	32	14.41%	50.00%
* P*^*1*^	-	-	-	< 0.001^b^	-
* P*^*2*^	-	-	-	0.008^b^	0.02^b^

*P*^*1*^ indicates the comparison between the BI-RADS classification and the combined model

*P*^*2*^ indicates the comparison of diagnostic performance before and after BI-RADS classification adjustment based on the combined model

^a^ Indicates evaluation limited to BI-RADS 4a breast lesions

^b^ indicates *p* < 0.05

## Discussion

This study extracted radiomics features from ultrasound images of breast lesions to evaluate the performance of multiple machine learning models in distinguishing benign from malignant lesions. By integrating clinical ultrasound features, a combined predictive model was developed and validated across multiple datasets and patient subgroups. The model also showed potential value in BI-RADS reclassification and biopsy decision-making.

Early and accurate diagnosis of breast cancer is essential for improving prognosis [25]. However, conventional ultrasound diagnosis depends heavily on physician expertise, and the BI-RADS system, while standardized, remains limited in diagnostic certainty. In particular, BI-RADS category 4 often presents overlapping imaging features between benign and malignant lesions, leading to high false-positive rates [26]. Radiomics provides a more objective and quantitative approach by extracting high-dimensional features beyond visual interpretation. This study extracted radiomics features that included shape descriptors, first-order statistical features, and various texture-based high-order features. Among them, wavelet-transformed gray-level and texture features were the most common. For example, “wavelet.LLL_glszm_ZonePercentage” describes the uniformity of textures within the lesion, and “wavelet.LLL_glrlm_RunLengthNonUniformityNormalized” captures gray-level consistency. These features characterize the microstructural heterogeneity of breast tumors [27], information that is difficult to capture with conventional ultrasound [28].

After completing feature selection, we constructed predictive models using five machine learning algorithms. The LR model demonstrated consistent performance across all datasets. The AUCs were 0.83, 0.82, 0.81, and 0.82 in the training, internal test, external test, and prospective test sets, respectively. In all three test sets, the LR model significantly outperformed the other algorithms. This may be attributed to the linear combination of feature weights in LR, which helps prevent overfitting and is suitable for radiomics datasets with fewer but informative features. These findings are in line with previous research by Ye et al [29]. Therefore, the LR model was selected as the final radiomics model. Furthermore, SHAP analysis was applied to improve interpretability, clarifying both the overall and individual contributions of radiomics features to model predictions.

In addition to radiomics features, traditional clinical ultrasound indicators also play an important role in predicting breast lesion malignancy. In this study, age, tumor size, orientation, margin, and shape were identified as independent predictors, consistent with previous studies [30–32]. Notably, orientation, margin, and shape are standard ultrasound descriptors from the BI-RADS lexicon, whereas age and tumor size are objective clinical/measurement variables that may provide complementary information beyond imaging appearance. We did not use the final BI-RADS assessment category as an input feature because it represents a composite, reader-dependent conclusion and was evaluated separately for biopsy-decision analysis and comparison with our models. The predictive value of age may be related to hormone-driven structural changes in breast tissue, especially with aging and menopausal status [33, 34]. Although the clinical model achieved good performance, it was less effective than the radiomics model in capturing subtle imaging details, particularly for complex or ambiguous lesions.

To leverage the complementary strengths of both approaches, we constructed a combined model that incorporated radiomics scores with clinical and ultrasound factors. This model achieved consistently high AUCs across all datasets (0.92, 0.90, 0.92, and 0.93), significantly outperforming either model alone (all *p* < 0.01). NRI and IDI analyses confirmed that radiomics scores added discriminative value beyond clinical features. Furthermore, across most threshold probability ranges across all test sets, the combined model showed higher net benefit in decision-curve analysis than the radiomics and clinical models alone. These results highlight that radiomics captures complementary information, particularly wavelet-based texture features, which are not accessible through traditional clinical indicators [35–37]. Importantly, SHAP value analysis clarified the contribution of each variable to the model’s output. Clinicians can examine SHAP plots to observe how values of specific features influence the prediction for each individual case. This type of quantitative interpretability enhances clinical acceptance of the model and supports its future implementation in practice.

The combined model also performed well across patient subgroups, achieving balanced sensitivity and specificity in smaller lesions and younger patients, which is important for early detection and diagnosis of breast cancer. Prior studies have suggested that combining radiomics with conventional ultrasound enhances early diagnostic accuracy [17, 29, 35–37], but most were limited by small cohorts and insufficient validation. By leveraging multicenter data and prospective testing, this study ensured broader generalizability and robustness, with consistent performance across different hospitals and ultrasound systems.

Compared with senior radiologists, the combined model achieved significantly higher accuracy than BI-RADS at 4a as the cutoff, while comparable accuracy was observed at higher thresholds (4b or 4c), consistent with previous findings [38]. Importantly, model-assisted reclassification of BI-RADS categories suggested the potential to reduce biopsy recommendations for benign lesions and increased the proportion of malignant lesions among BI-RADS 4a cases. These findings suggest that the combined model may support biopsy decision-making and lesion risk stratification, pending further prospective clinical utility studies. However, both the clinical and combined models incorporated reader-derived ultrasound descriptors assessed by experienced radiologists (including orientation, margin, and shape); therefore, comparisons with BI-RADS assessment should not be interpreted as purely automated human-versus-machine comparisons.

This study has several limitations. First, the study population was highly selected because only lesions with pathological confirmation were included, resulting in a relatively high prevalence of malignancy and a cohort that is not fully representative of routine diagnostic ultrasound practice. This cohort composition may have influenced the apparent model performance, decision-curve analysis findings, and biopsy reclassification estimates. Therefore, these results should be interpreted with caution, as they may not directly translate to lower-prevalence real-world settings. Second, although data from multiple centers were included, variations in ultrasound equipment and protocols across centers may have influenced image quality and model performance, suggesting the need for standardized acquisition in future studies. Finally, the radiomics features were manually extracted. In the future, deep learning-based automatic feature extraction methods may be considered to reduce manual intervention during feature selection and improve the efficiency and automation of the modeling process.

## Conclusions

In summary, this study developed and validated a combined model integrating radiomics and clinical features for breast cancer diagnosis. Multicenter and prospective validation confirmed its accuracy, robustness, and interpretability. By showing improved diagnostic performance and interpretable prediction patterns, the model may support breast lesion risk stratification and biopsy decision-making, pending further prospective clinical utility studies.

## Supplementary information

ELECTRONIC SUPPLEMENTARY MATERIAL

## Data Availability

The datasets used and analyzed during the current study are available from the corresponding author on reasonable request.

## Abbreviations

AUC: Area under the curve
BI-RADS: Breast Imaging Reporting and Data System
DCA: Decision curve analysis
IDI: Integrated discrimination improvement
KNN: K-nearest neighbor
LR: Logistic regression
MLP: Multi-layer perceptron
MRI: Magnetic resonance imaging
NRI: Net reclassification improvement
RF: Random Forest
ROC: Receiver operating characteristic
ROI: Region of interest
SHAP: Shapley additive explanations
SVM: Support vector machine
