# Supplementary material for: Development and validation of an interpretable ultrasound radiomics model for benign and malignant classification of breast lesions: a multicenter large-sample study
Source: Insights Imaging. 2026 Jun 25;17:174. doi: 10.1186/s13244-026-02344-y (PMC13294407; doi:10.1186/s13244-026-02344-y)
Supplement: Supplementary file 1 — ELECTRONIC SUPPLEMENTARY MATERIAL [file 13244_2026_2344_MOESM1_ESM.pdf]

Development and Validation of an Interpretable Ultrasound Radiomics Model for Benign and Malignant Classification of Breast Lesions: A Multicenter Large-sample Study

ELECTRONIC SUPPLEMENTARY MATERIAL

Supplementary Material S1. **Radiomics feature selection process.**

First, radiomics features with an ICC below 0.80 were excluded. The remaining features were then normalized across the training set, internal test set, external test set, and prospective test set using Z-score transformation. This normalization ensured that all data were scaled to the same range, reducing the influence of different imaging settings and operators on ultrasound images and improving the comparability of radiomics features. Next, redundancy analysis was performed on the features extracted from the training set. Pearson correlation analysis was applied to features following a normal distribution, while Spearman correlation analysis was used for non-normally distributed features. Features with a correlation coefficient greater than 0.90 were removed to eliminate redundancy. To minimize overfitting and selection bias in radiomics features, the least absolute shrinkage and selection operator (LASSO) regression method was employed for feature selection. A five-fold cross-validation approach was used to determine the optimal penalty parameter Log(λ) based on the criterion of minimizing cross-validation error. In LASSO regression,

as the penalty parameter λ increases, the coefficients of features gradually shrink toward zero. The optimal λ value was selected as the one that minimized the cross-validation error, and ultimately, only radiomics features with nonzero coefficients in the training set were retained.

Supplementary Material S2. **Radiomics feature selection results.**

A total of 851 quantitative radiomics features were extracted from each image and its corresponding ROI. These features included 14 morphological features, 162 first-order statistical features, 216 gray level cooccurrence matrix (GLCM) features, 126 gray level dependence matrix (GLDM) features, 144 gray level run length matrix (GLRLM) features, 144 gray level size zone matrix (GLSZM) features, and 45 neighboring gray tone difference matrix (NGTDM) features. Features with both intra- and inter-observer ICC values below 0.80 were excluded, resulting in 482 remaining radiomics features. Subsequently, redundancy analysis and LASSO regression were applied, further reducing the number of features to 12 (**Fig. 1**). The final selected features included:

（1）original_shape_Elongation，

（2）wavelet.LLH_firstorder_10Percentile，

（3）wavelet.LHH_firstorder_10Percentile，

（4）wavelet.LHH_firstorder_Skewness，

（5）wavelet.HLL_firstorder_90Percentile，

（6）wavelet.HLH_firstorder_RobustMeanAbsoluteDeviation，

（7）wavelet.HLH_glszm_GrayLevelNonUniformity，

（8）wavelet.HHH_glszm_GrayLevelNonUniformity，

（9）wavelet.LLL_firstorder_RobustMeanAbsoluteDeviation，

（10）wavelet.LLL_firstorder_TotalEnergy，

（11）wavelet.LLL_glrlm_RunLengthNonUniformityNormalized，

（12）wavelet.LLL_glszm_ZonePercentage

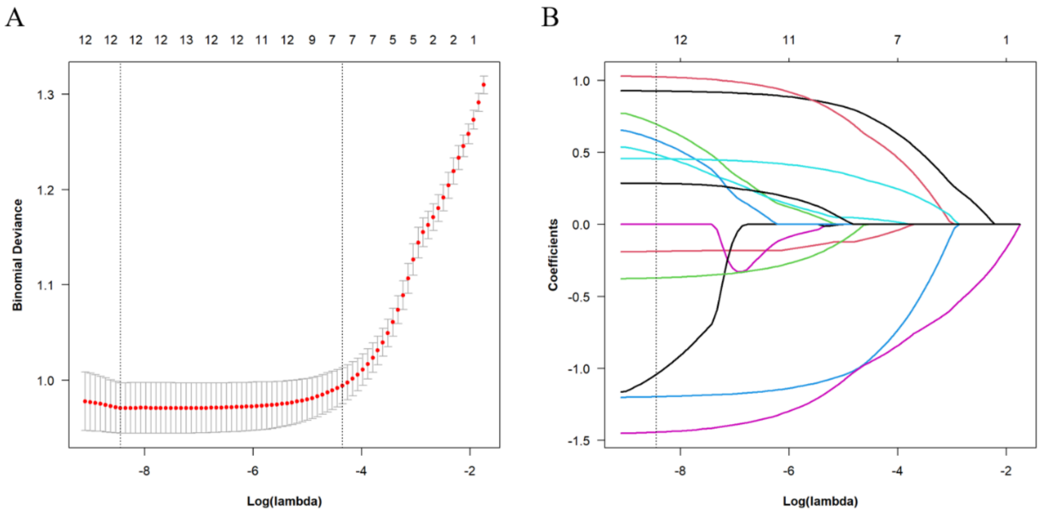

Fig. 1. Selection of radiomics features related to benign and malignant breast lesions using LASSO regression analysis in the training set. (A) generation of the optimal penalty coefficient λ for the radiomics features in LASSO regression analysis using five-fold cross-validation and the minimum criterion; (B) LASSO coefficient curve for feature selection.

Supplementary Table S1. Enrollment period and eligible sample size by center.

Center	Study design	Enrollment period	Eligible patients
Hospital 1	Retrospective	September 1, 2019 - March 9, 2024	1102
Hospital 1	Prospective	March 10, 2024 - September 30, 2024	267
Hospital 2	Retrospective	February 1, 2012 - December 31, 2022	919
Hospital 3	Retrospective	January 1, 2020 - November 30, 2022	391
Hospital 4	Retrospective	April 1, 2021 - October 31, 2022	211
Hospital 5	Retrospective	June 1, 2020 - April 30, 2021	120
Hospital 6	Retrospective	October 1, 2021 - October 31, 2022	81
Hospital 7	Retrospective	October 1, 2022 - July 31, 2023	25
Hospital 8	Retrospective	March 1, 2022 - November 30, 2022	26

Supplementary Table S2. Univariate and multivariate logistic regression analysis of benign and malignant breast lesions in the training set.

Characteristic	Univariate Analysis		Multivariate Analysis			
			Clinical Model		Combined model	
	OR (95% CI)	*P* value	OR (95% CI)	*P* value	OR (95% CI)	*P* value
Age	1.08 (1.07, 1.09)	<0.001*	1.07 (1.06, 1.09)	<0.001*	1.07 (1.06, 1.08)	<0.001*
Tumor size	1.42 (1.29, 1.56)	<0.001*	1.41 (1.25, 1.60)	<0.001*	1.24 (1.09, 1.42)	0.002*
Tumor location (Right)	0.94 (0.77, 1.15)	0.54	NA	NA	NA	NA
Orientation (Nonparallel)	4.52 (3.02, 6.76)	<0.001*	3.21 (2.01, 5.13)	<0.001*	2.41 (1.45, 4.00)	<0.001*
Margin (Non-circumscribed)	8.01 (6.40, 10.03)	<0.001*	3.75 (2.84, 4.94)	<0.001*	3.71 (2.71, 5.07)	<0.001*
Shape (Irregular)	17.51 (12.76, 24.02)	<0.001*	8.25 (5.66, 12.03)	<0.001*	6.01 (3.98, 9.08)	<0.001*
Echotexture						
hypoecho	Ref					
Isoechoic	0.21 (0.06, 0.81)	0.02	NA	NA	NA	NA
Hyperechoic	0.43 (0.10, 1.91)	0.27	NA	NA	NA	NA
Complex cystic and solid	0.73 (0.45, 1.19)	0.21	NA	NA	NA	NA
Heterogeneous	1.36 (0.89, 2.07)	0.15	NA	NA	NA	NA
Posterior features						
None	Ref					
Shadowing	2.96 (2.25, 3.90)	<0.001*	NA	NA	NA	NA
Enhancement	0.97 (0.74, 1.27)	0.82	NA	NA	NA	NA
Combined pattern	2.68 (1.73, 4.13)	<0.001*	NA	NA	NA	NA
Radiomics score	192.33 (115.07, 321.45)	<0.001*	NA	NA	91.24 (48.74, 170.79)	<0.001*

Note: * indicates *P* < 0.05.

Abbreviations: CI, confidence interval; NA, not applicable; OR, odds ratio; Ref, reference value.

Supplementary Table S3. Diagnostic performance evaluation of the radiomics model, clinical model, and combined model across each dataset.

	AUC (95% CI)	ACC	SEN	SPE	PPV	NPV
Training set						
Radiomics model	0.83 (0.81-0.85)	76.48%	81.27%	68.13%	81.65%	67.58%
Clinical model	0.87 (0.86-0.89)	81.46%	83.60%	77.72%	86.75%	73.09%
Combined model	**0.92* (0.90-0.93)**	84.95%	86.11%	82.93%	89.80%	77.39%
Internal test set						
Radiomics model	0.82 (0.79-0.85)	76.66%	83.05%	64.68%	81.50%	67.08%
Clinical model	0.85 (0.83-0.88)	79.56%	83.90%	71.43%	84.62%	70.31%
Combined model	**0.90* (0.87-0.92)**	82.87%	85.38%	78.18%	87.99%	74.06%
External test set						
Radiomics model	0.81 (0.78-0.85)	77.54%	90.64%	52.22%	75.50%	83.93%
Clinical model	0.87 (0.84-0.90)	79.70%	82.33%	75.56%	84.12%	73.12%
Combined model	**0.92* (0.89-0.94)**	85.96%	86.93%	84.44%	89.78%	80.42%
Prospective test set						
Radiomics model	0.82 (0.77-0.87)	81.65%	99.48%	35.14%	80.00%	96.30 %
Clinical model	0.86 (0.81-0.90)	81.27%	87.05%	66.22%	87.05%	66.22 %
Combined model	**0.93* (0.89-0.96)**	84.27%	88.60%	72.97%	89.53%	71.05 %

Note: * indicates that the AUC of this model is significantly higher than those of the other models according to the DeLong test (*P* < 0.05).

Abbreviations: Acc, accuracy; AUC, area under the receiver operating characteristic curve; CI, confidence interval; NPV, negative predictive value; PPV, positive predictive value; SEN, sensitivity; SPE, specificity.

Supplementary Table S4. Diagnostic performance of the combined model in different patient subgroups.

	AUC (95% CI)	ACC	SEN	SPE	PPV	NPV
Training set						
≤ 2 cm	0.91 (0.89-0.93)	83.47%	80.69%	86.74%	87.77%	79.21%
> 2 cm	0.91 (0.90-0.93)	86.16%	89.46%	78.00%	90.97%	74.91%
< 40 years	0.87 (0.84-0.90)	81.82%	63.56%	89.57%	72.12%	85.27%
≥ 40 years	0.91 (0.89-0.92)	85.91%	88.90%	77.45%	91.78%	71.12%
Internal test set						
≤ 2 cm	0.88 (0.84-0.92)	80.57%	80.65%	80.47%	83.33%	77.44%
> 2 cm	0.90 (0.87-0.93)	84.35%	87.70%	75.81%	90.26%	70.68%
< 40 years	0.88 (0.81-0.92)	81.48%	67.31%	88.18%	72.92%	85.09%
≥ 40 years	0.87 (0.84-0.90)	83.57%	88.04%	70.42%	89.76%	66.67%
External test set						
≤ 2 cm	0.92 (0.88-0.96)	84.04%	80.41%	87.07%	83.87%	84.17%
> 2 cm	0.89 (0.85-0.93)	87.60%	90.32%	79.69%	92.82%	73.91%
< 40 years	0.90 (0.93-0.95)	82.46%	61.77%	91.25%	75.00%	84.88%
≥ 40 years	0.90 (0.87-0.93)	87.11%	90.36%	79.00%	91.46%	76.70%

Prospective test set						
≤ 2 cm	0.94 (0.88-0.98)	83.93%	89.04%	74.36%	86.67%	78.38%
> 2 cm	0.92 (0.86-0.96)	84.52%	83.33%	71.43%	91.38%	64.10%
< 40 years	0.88 (0.74-0.96)	76.19%	64.71%	84.00%	73.33%	77.78%
≥ 40 years	0.93 (0.89-0.96)	85.78%	90.91%	67.35%	90.91%	67.35%

Abbreviations: Acc, accuracy; AUC, area under the receiver operating characteristic curve; CI, confidence interval; NPV, negative predictive value; PPV, positive predictive value; SEN, sensitivity; SPE, specificity.

Supplementary Table S5. Evaluation of the performance improvement of predictive models through NRI and IDI indices.

Characteristic	Combined model vs. Clinical model	*P* value
Training set		
Categorical NRI (95% CI)	0.13（0.08, 0.17）	<0.001*
Continuous NRI (95% CI)	1.06（0.98, 1.15）	<0.001*
IDI（95% CI）	0.11（0.09, 0.13）	<0.001*
Internal test set		
Categorical NRI (95% CI)	0.10（0.03, 0.17）	0.004*
Continuous NRI (95% CI)	0.98（0.84, 1.11）	<0.001*
IDI（95% CI）	0.09（0.07, 0.12）	<0.001*
External test set		
Categorical NRI (95% CI)	0.18（0.10, 0.26）	<0.001*
Continuous NRI (95% CI)	0.97（0.81, 1.14）	<0.001*
IDI（95% CI）	0.13（0.09, 0.17）	<0.001*
Prospective test set		<0.001*
Categorical NRI (95% CI)	0.15（0.02, 0.28）	0.022*
Continuous NRI (95% CI)	1.13（0.90, 1.36）	<0.001*
IDI（95% CI）	0.17（0.11, 0.24）	<0.001*

Note: * indicates $P < 0.05$.

Abbreviations: CI, confidence interval; IDI, integrated discrimination improvement; NRI, net reclassification improvement. *$p < 0.05$.

Supplementary Table S6. Comparison of the diagnostic performance between the combined model and BI-RADS classification.

	External test set			Prospective test set		
	Accuracy	Sensitivity	Specificity	Accuracy	Sensitivity	Specificity
Combined model	85.96%	86.93%	84.44%	84.27%	88.60%	72.97%
BI-RADS (Classification A)	74.08%	99.65%	33.89%	76.40%	98.96%	17.57%
BI-RADS (Classification B)	85.53%	97.53%	66.67%	86.89%	95.34%	64.87%
BI-RADS (Classification C)	84.02%	79.51%	91.11%	85.39%	84.46%	87.84%
P^1	<0.001*	<0.001*	<0.001*	0.01*	<0.001*	<0.001*
P^2	0.91	<0.001*	<0.001*	0.36	0.01*	0.24
P^3	0.42	0.01*	0.0501	0.79	0.26	0.03*

Note: Classification A: BI-RADS 4a used as the benign/malignant cutoff; Classification B: BI-RADS 4b used as the cutoff; Classification C: BI-RADS 4c used as the cutoff. P^1: comparison between the combined model and BI-RADS (Classification A); P^2: comparison between the combined model and BI-RADS (Classification B); P^3: comparison between the combined model and BI-RADS (Classification C).

* Indicates $P < 0.05$.

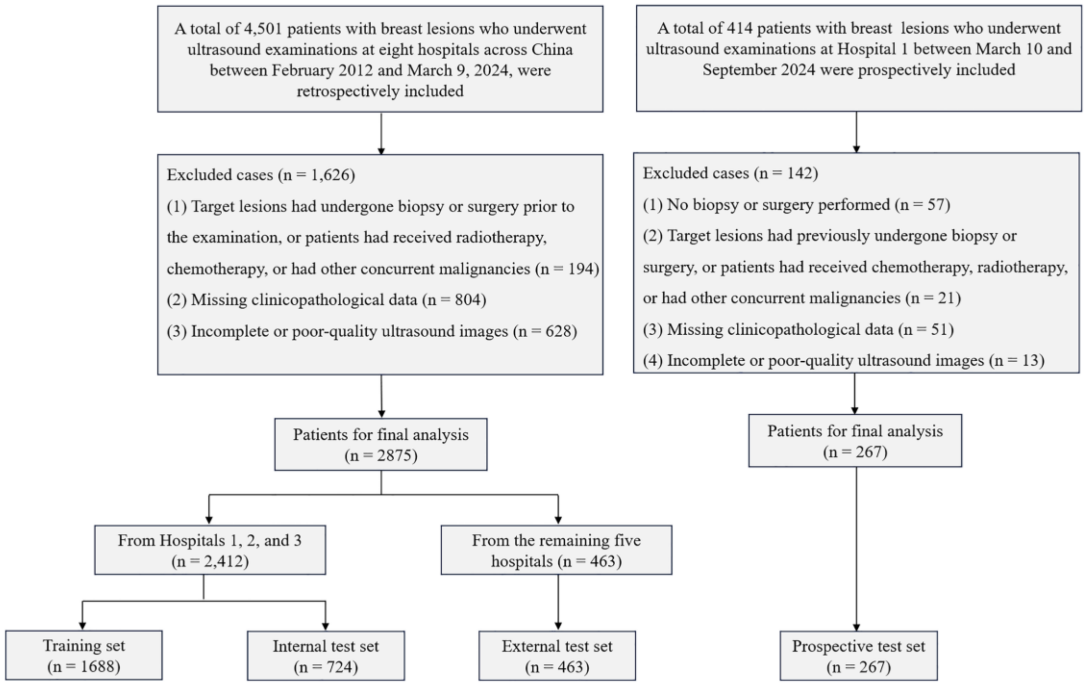

Supplementary Fig. S1. Flow diagrams of study populations.

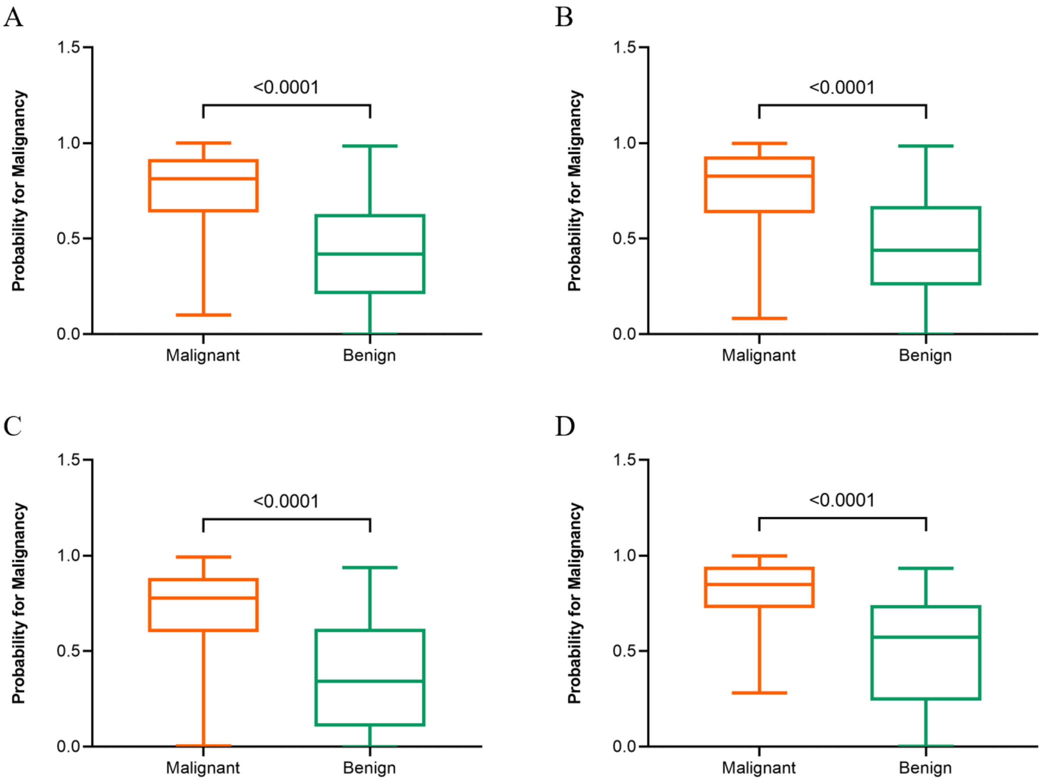

Supplementary Fig. S2. Discriminatory performance of the LR model in distinguishing benign and malignant breast lesions across four datasets: (A) training set, (B) internal test set, (C) external test set, (D) prospective test set.

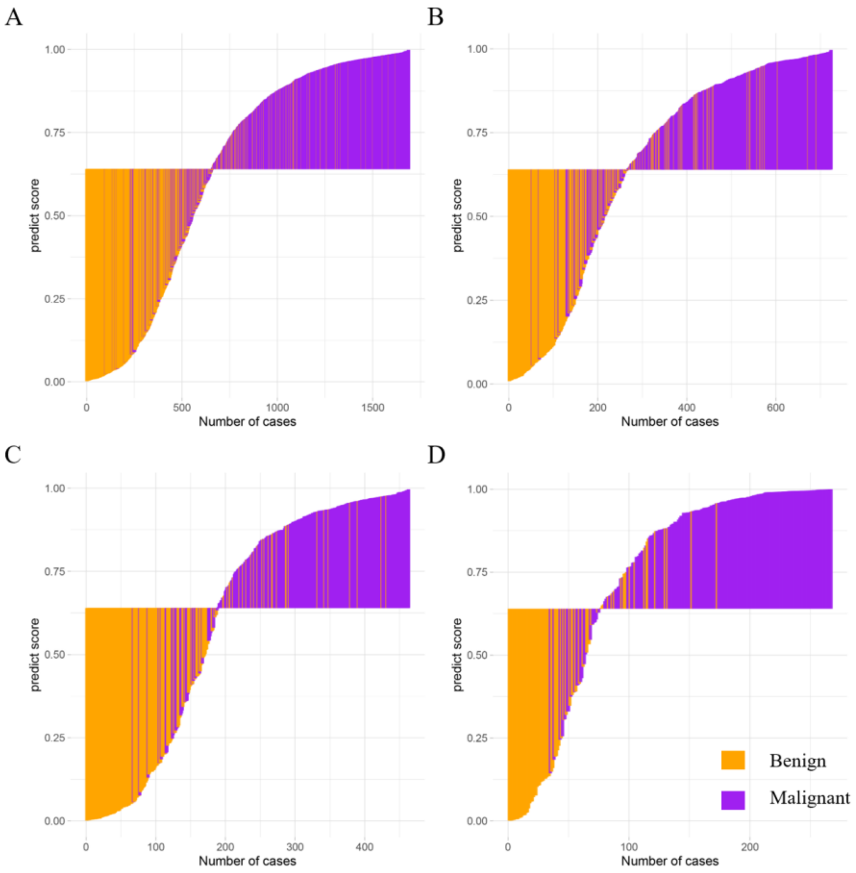

Supplementary Fig. S3. Distribution of predicted risk probabilities by the combined model in different datasets. (A) training set; (B) internal test set; (C) external test set; (D) prospective test set.

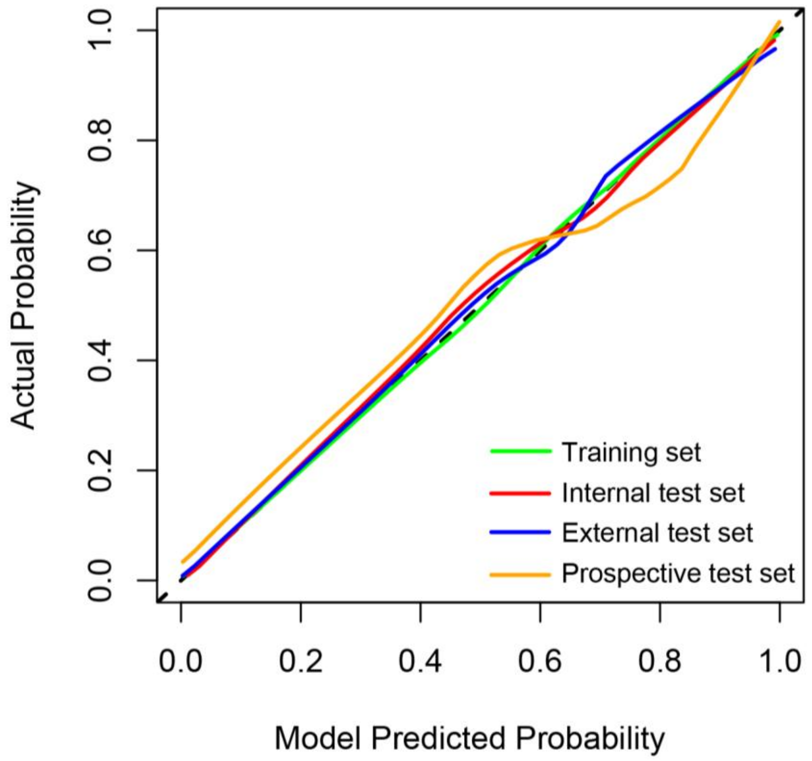

Supplementary Fig. S4. Calibration curves of the combined model in different datasets.
